# Supplementary material for: Identification of photoperiod-induced specific miRNAs in the adrenal glands of Sunite sheep (Ovis aries)
Source: Front Vet Sci. 2022 Jul 22;9:888207. doi: 10.3389/fvets.2022.888207 (PMC9354845; doi:10.3389/fvets.2022.888207)
Supplement: Supplementary file 1 [file Data_Sheet_1.ZIP › Supplementary file/miRDeep2 file.zip!/miRDeep2 file/Novel_Structure/SP42A2/Novel_411.pdf]

Provisional ID	:	NC_019464.2_17685
Score total	:	0.7
Score for star read(s)	:	-1.3
Score for read counts	:	0
Score for mfe	:	0.4
Score for randfold	:	1.6
Score for cons. seed	:	
Total read count	:	54
Mature read count	:	54
Loop read count	:	0
Star read count	:	0

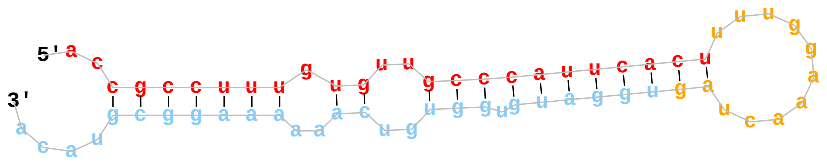

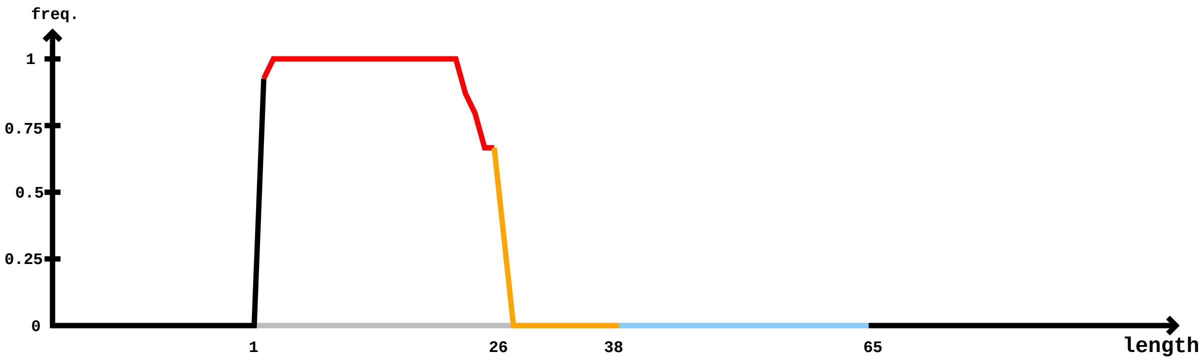

Mature **Star**

```
5'- auuccuuaugcgguuuucuuaccgccuuuguguugcccauucacuuuuggaaacuaguggauguggugucaaaaaaggcguacacaaaacacuuucgcagcuuuuuuuccugccc   -3'   exp
    ............(((((.....(((((((.((..(((((((((((...........))))))))).)))..))..))))))).....))))).......((((.........))))..    reads      mm         sample
    ....................accgccuuuguguugcccauu..........................................................................    7          0          seq
    ....................accgccuuuguguugcccauuc.........................................................................    4          0          seq
    ....................accgccuuuguguugcccauuca........................................................................    6          0          seq
    ....................accgccuuuguguugcccauucacu......................................................................    28         0          seq
    ....................accgccuuuguguugcccauucacuu.....................................................................    5          0          seq
    .....................ccgccuuuguguugcccauuca........................................................................    1          0          seq
    .....................ccgccuuuguguugcccauucacu......................................................................    2          0          seq
    .....................ccgccuuuguguugcccauucacuu.....................................................................    1          0          seq
```
